# Magnetic Resonance Imaging-Based Prediction of the Relationship between Whiplash Injury and Temporomandibular Disorders

**DOI:** 10.3389/fneur.2017.00725

**Published:** 2018-01-09

**Authors:** Yeon-Hee Lee, Kyung Mi Lee, Q-Schick Auh, Jyung-Pyo Hong

**Affiliations:** ^1^Orofacial Pain and Oral Medicine, Kyung Hee University Dental Hospital, Seoul, South Korea; ^2^Radiology, Kyung Hee University College of Medicine, Kyung Hee University Hospital, Seoul, South Korea

**Keywords:** whiplash injury, temporomandibular disorder, macrotrauma, magnetic resonance imaging, neck pain

## Abstract

**Purpose:**

Whiplash injury can cause internal derangement of the temporomandibular joint (TMJ) and lead to temporomandibular disorders (TMDs). Our aim was to evaluate whether the initial clinical findings in TMD patients with whiplash injury are correlated with their magnetic resonance imaging (MRI) characteristics.

**Materials and methods:**

This case–control study involved 219 patients (135 women, 84 men; mean age: 37.84 years) who visited our orofacial pain clinic with TMD; TMD was diagnosed using the diagnostic criteria for TMD Axis I. Patients were categorized into three groups based on the presence and type of macrotrauma: in the “wTMD” group, patients had suffered whiplash injury; patients in the “pTMD” group had post-traumatic TMD; the “iTMD” group comprised patients who had presented with TMD symptoms and had sustained no macrotrauma. We investigated the presence of disk displacement, effusion, disk deformity, and condylar degeneration, and changes in the lateral pterygoid muscle (LPM). To evaluate the severity of TMD pain and objectively analyze symptoms, we used a visual analog scale (VAS), palpation index (PI), neck PI, dysfunction index, and craniomandibular index (CMI).

**Results:**

The VAS scores, and the severity indexes of the TMD including PI, neck PI, and CMI were highest in the wTMD patients. Atrophy of the LPM was most commonly seen in the wTMD group, as was disk deformity. In wTMD patients only, VAS score was significantly correlated with stress; it was correlated with headache in wTMD and iTMD patients. The clinical symptoms of TMD were not correlated with MRI findings in the wTMD group. However, alterations in the LPM were strongly correlated with disk displacement.

**Conclusion:**

If clinicians recognize alterations in the LPM and disk displacement in the TMJ, they will better understand the clinical symptoms and pathophysiology of TMD with whiplash injury. Whiplash injury may lead to TMD *via* different mechanisms from other macrotraumas.

## Introduction

**Figure 1:**
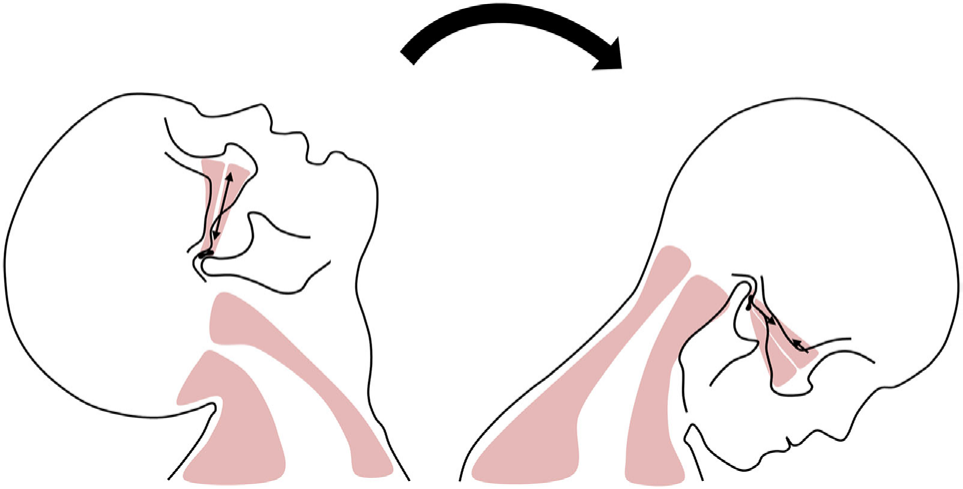
Possible mechanism of temporomandibular disorder evoked from whiplash injury. On extension, the temporomandibular joint (TMJ) elongated and abrupt mouth opening can occur. On flexion, the TMJ is compressed and lateral pterygoid muscle’s spasm can occur.

Whiplash injury results from an acceleration–deceleration mechanism of energy transfer to the neck predominantly in motor vehicle accidents (1). Whiplash is associated with a wide variety of clinical manifestations, including neck pain, neck stiffness, problems with psychological distress, and temporomandibular disorders (TMDs) (Figure 1) (2, 3). Previous reports using magnetic resonance imaging (MRI) have reported that a high percentage of TMD patients have abnormal effusion, disk displacement, and alterations in the thickness of the lateral pterygoid muscle (LPM) (4, 5). In one MRI study involving patients who had TMD symptoms after whiplash, 88% of all participants had a whiplash injury-related temporomandibular joint (TMJ) abnormality, such as disk displacement (56%), or abnormal joint fluid or edema (65%) of the TMJ (6). Importantly, current data indicate that up to 50% of people who experience a whiplash injury will remain disabled by their condition and never fully recover (7). In addition, whiplash injury entails substantial secondary economic costs, such as medical care for persistent disability and reduced work productivity, as well as primary personal costs.

Although previous studies have shown that whiplash injury is associated with TMD symptoms, the pathophysiology and mechanism of this relationship are still not well understood. Almost all hypotheses regarding the mechanism of rear impact-induced injury are based on kinematics (8). Specifically, the muscular trauma resulting from rear impact acceleration is often explained in terms of neck hyperextension and hyperflexion. TMD is a set of conditions affecting the masticatory muscles or joints; pain is the primary characteristic of this condition (9). Injury to the head and neck can occur primary or secondary to eccentric contraction or spasm. In particular, the LPM can be injured in this way, leading to internal derangement and TMD, which in turn influences rotation and translation of the disk and condyle (10). Nonetheless, recent biomechanical investigations and MRI studies have not been able to explain fully the TMD symptoms associated with disk-related injury.

Relatedly, whiplash injury is not a homogenous condition, and persistent whiplash injury can elevate psychological distress, probably because of ongoing pain and disability (11). The following features can be used to differentiate patients with whiplash injury: persistent moderate/severe symptoms, widespread hypersensitivity, chronic pain, and disability. These symptoms are reportedly due to sensitization of the central nervous system’s nociceptive pathways, or to changes in endogenous descending pain modulation mechanisms (12). Furthermore, higher levels of pain and disability in acute whiplash injury are a sign of poor outcome. For this reason, it is important that psychological factors be taken into account when measuring pain intensity.

The present study addressed the correlation between TMD clinical symptoms and MRI findings in TMD patients with whiplash injury, those with another macrotrauma, and those with no trauma. We also investigated how this correlation differed among these three groups. By comparing these groups, we aimed to ascertain whether TMD is related to whiplash injury. Additionally, using logistic regression analysis, we investigated the prospective longitudinal development of TMD following whiplash injury.

## Materials and Methods

### Subjects and Demographic Data

The present study was carried out in accordance with the Declaration of Helsinki and was approved by the institutional review board of our institute. This retrospective case–control study involved 219 consecutive patients (135 women, 84 men; mean age: 37.84 ± 16.40 years) with TMD; they were diagnosed using the diagnostic criteria for TMD Axis I (13). We identified patients who had retrospectively reviewing all MRI of TMJ and TMJ reports from January 2000 through 2016. The inclusion criteria were as follows: no history of neck pain prior to the traumatic incident, no treatment of the current episode other than medication, no history of direct trauma to the jaw before or during the accident—the patients suffered macrotrauma only, and no history of a TMJ disorder prior to the present TMD symptoms. The exclusion criteria were: serious injury such as unstable multiple trauma and facial fracture, rheumatoid disease, previous injury, psychological problems, pregnancy, psychiatric or neurological disorder unrelated to the trauma, and musculoskeletal disorder predating injury.

The patients were divided into three groups based on the type and the presence of macrotrauma history: in the “wTMD” group (*n* = 76), the patients had experienced whiplash injury and had no TMD symptoms before the injury; patients in the “pTMD” group (*n* = 58) had post-traumatic TMD, but had experienced macrotrauma other than whiplash injury; the “iTMD” group (*n* = 85) comprised patients who had presented with idiopathic/non-traumatic TMD symptoms without any history of head/neck trauma. Table 1 shows the sex and age distribution among the three groups (Table 1).

**Table 1:** Demographic description and comparison of the mean and SDs of variables.

Total (*n* = 219)	wTMD group (*n* = 76)	pTMD group (*n* = 58)	iTMD group (*n* = 85)	*p*-Value	*Post hoc* analysis
**Sex distribution**					
Male, *n* (%)	22 (28.95)	33 (56.90)	33 (34.12)	0.003	wTMD–pTMD, pTMD–iTMD
Female, *n* (%)	54 (71.05)	25 (43.10)	56 (65.88)	0.311	n.s.
Age (mean ± SD)	33.29 ± 14.15	35.78 ± 17.00	37.95 ± 17.84	0.471	n.s.
VAS (mean ± SD)	6.88 ± 1.94	5.74 ± 2.25	5.32 ± 2.90	<0.001	wTMD > pTMD = iTMD
**TMD indexes**					
Neck PI (mean ± SD)	0.46 ± 0.50	0.10 ± 0.31	0.15 ± 0.36	<0.0001	wTMD > pTMD = iTMD
PI (mean ± SD)	0.29 ± 0.23	0.15 ± 0.15	0.17 ± 0.16	<0.0001	wTMD > pTMD = iTMD
DI (mean ± SD)	0.51 ± 0.27	0.46 ± 0.24	0.43 ± 0.23	0.073	n.s.
CMI (mean ± SD)	0.40 ± 0.23	0.31 ± 0.14	0.30 ± 0.17	0.001	wTMD > iTMD
**Mouth opening (mm)**					
CMO (mean ± SD)	34.68 ± 11.29	33.48 ± 12.75	35.19 ± 10.07	0.672	n.s.
MMO (mean ± SD)	40.67 ± 11.26	38.97 ± 12.97	40.26 ± 10.10	0.674	n.s.
**Eccentric movement (mm)**					
Protrusion (mean ± SD)	4.58 ± 2.43	5.00 ± 3.27	4.82 ± 2.59	0.669	n.s.
Rt. laterotrusion (mean ± SD)	6.83 ± 3.15	6.48 ± 3.99	6.96 ± 3.22	0.705	n.s.
Lt. laterotrusion (mean ± SD)	7.12 ± 2.94	7.28 ± 4.65	7.85 ± 3.82	0.443	n.s.

*wTMD–pTMD, when the proportion of the presence of variable was significantly different between wTMD and pTMD groups; wTMD–iTMD, when the proportion of the presence of variable was significantly different between wTMD and iTMD groups; pTMD–iTMD, when the proportion of the presence of variable was significantly different between pTMD and iTMD groups; n.s., when there was no significant difference between groups; VAS, visual analog scale; TMD, temporomandibular disorder; PI, palpation index; DI, dysfunction index; CMI, craniomandibular index; CMO, comfortable mouth opening; MMO, maximum mouth opening; SD, standard deviation*.

### MRI Acquisition

All patients underwent MRI examination of the bilateral TMJ; images were taken using a 1.5 T MRI system (Genesis Signa; GE Medical System), with a 6-cm × 8-cm diameter surface coil. All scans involved sagittal oblique sections of 3 mm or less, a 15-cm field of view, and a 256 × 224 matrix. T2-weighted images (T2WIs) were obtained using a 2,650/82 TR/TE sequence; T1-weighted images (T1WIs) were obtained using a 650/14 TR/TE sequence; and proton density images were obtained using a 2,650/82 TR/TE sequence. Spin-echo sagittal MR images were planned on the axial localizer images.

TMJs were imaged in the sagittal and coronal planes to determine the presence of internal derangement, effusion, disk deformity, condylar degeneration, or LPM alteration. Disk position in the oblique sagittal plane was determined in the closed- and open-mouth positions. The images were interpreted by two independent observers; both were experts in head and neck MRI.

### Validation of MRI Findings (Figure 2)

#### Evaluation of TMJ Internal Derangement

When the disk was located above the condyle, it was regarded as being in the normal position. Anterior disk displacement was identified when the disk was located anterior to the condyle in the closed-mouth position. Anterior disk displacement with reduction (ADDWR) was confirmed when the normal condyle–disk relationship was restored in the open-mouth position. Finally, when the disk remained anterior to the condyle in the open-mouth position, anterior disk displacement without reduction (ADDWoR) was confirmed.

**Figure 2:**
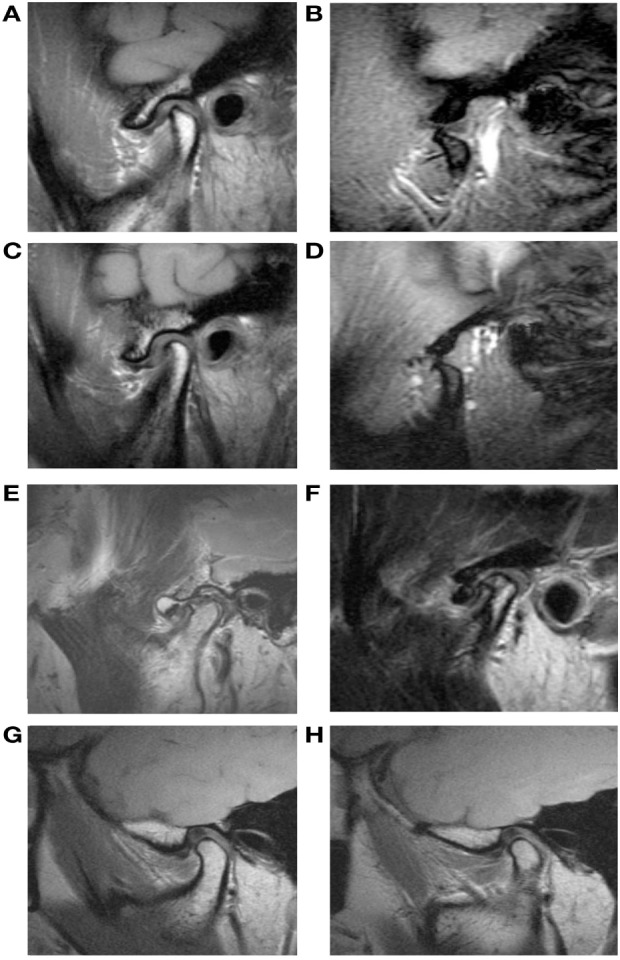
Magnetic resonance imaging (MRI) features of the abnormal temporomandibular joint disk and lateral pterygoid muscle (LPM). **(A,B)** Anterior disk displacement with reduction. Sagittal oblique gradient-echo T1-weighted image with closed-mouth position shows an anterior displaced disk **(A)** and MRI with open-mouth position shows that the disk has returned to its normal position between the condyle and the temporal bone **(B)**. **(C,D)** Anterior disk displacement without reduction. MR image shows a disk displaced from its normal position in closed-mouth position **(C)** and the disk remains displaced from its normal location in the open-mouth view **(D)**. **(E)** Sagittal T2-weighted image (T2WI) shows a round shape displaced disk and arthritis of mandibular condyle. **(F)** T2WI shows clearly delineated articular fluid collection with hyperintensity. Sagittal proton density image shows fatty and atrophic change of right LPM **(G)** when compared with contralateral normal LPM **(H)**.

#### Joint Effusion

T2WI was used to assess joint effusion. If effusion was present around the disk, or if it occupied the superior and/or inferior joint space, we determined that effusion was present.

#### Disk Deformity

When the disks were folded, thinned, lengthened, perforated, or had thick intermediate zones in the proton density images and T2WIs, we considered disk deformity to be present.

#### Condylar Degeneration

When articular cartilage degeneration and morphological changes such as bony erosive change and bony contour deformity were observed in T1WIs or T2WIs, we considered condylar degeneration to have occurred.

We evaluated muscle atrophy, contracture, and morphological alterations in the LPM. The LPM was considered atrophic when fatty replacement tissue with a high-intensity signal was present across wide areas of the muscle on T1WIs and T2WIs. In the same images, contracture of the LPM presented as fibrosis and appeared as an area of low-density signal.

### Validation of Clinical TMD Signs and Symptoms

#### Parafunctional Behaviors and TMD Symptoms

We assessed the self-reported presence of parafunctional activities using the oral behaviors checklist, which includes jaw-related behaviors such as clenching the teeth and bruxism (14). The clinical information for fulfilling the Axis I diagnostic criteria is obtained from the specified examination protocol that assessed TMD symptoms (13). The presence of headache attributed to TMD was asked based upon the criteria of the International Classification of Headache Disorders (15). Each parameter was reported as a binary answer (yes/no) in each patient.

#### Pain Characteristics

Pain duration in the face, jaw, jaw joint, and temple was reported in days. Pain intensity was measured in the jaw and face, as well as in the neck, using a visual analog scale (VAS) that was labeled from 0 (no pain) to 10 (pain as bad as you can imagine).

#### Palpation Index (PI), Neck PI, Dysfunction Index (DI), and Craniomandibular Index (CMI)

The PI, DI, and CMI are reliable scoring systems that allow TMD to be objectively assessed; in particular, they analyze the severity of painful TMDs. In each subject, we palpated 20 intra- and extra-oral muscle sites, including 3 in the neck and 4 in the TMJ. For each site, a binary answer (yes/no) was given. To calculate the PI, we added up all the positive answers and divided it by the number of events (16). To investigate the intensity of neck pain further, we calculated the neck PI, which was defined as the number of positive answers in the palpation of neck muscles divided by the number of events. The DI was defined as the number of positive answers regarding mandibular movements, joint noise, and joint capsule sensitivity divided by the number of events. To calculate the CMI, we added the DI to the PI and divided by 2. Using these four indexes, we quantified the clinical symptoms of TMD.

#### Mandibular Range of Motion

The vertical range of motion was measured (in millimeters) using a ruler; more specifically, the comfortable mouth opening (CMO) without pain and the unassisted maximum mouth opening (MMO) with pain were quantified. Eccentric movement was understood to mean the range of mandibular protrusion and lateral excursion. All measurements were made between the incisal edges of the maxillary central and the mandibular right central teeth. The normal extent of jaw opening ranges from 40 to 60 mm, depending on the age, gender, and height of the participant (17).

### Statistical Methods

To carry out descriptive data analysis, we obtained the absolute and percentage distributions of all nominal and categorical variables, as well as the mean, SD, and variation coefficient (for numerical variables). To analyze the data, we used the χ^2^ test for proportions’ equality, Fisher’s exact test, and the Bonferroni test. Spearman’s correlation analysis was used to determine the correlations between the variables. To compare the mean values among the three groups, we used analysis of variance and the Tukey’s paired comparison test. To estimate time-dependent changes in the MRI findings, we performed a logistic regression analysis. Statistical significance was established at *p* < 0.05. The data were analyzed using IBM’s Statistical Package for the Social Sciences (SPSS) version 20.0 (SPSS Inc., Chicago, IL, USA).

## Results

### Pain Intensity Analysis among the Three Groups

The mean VAS score in the wTMD group was 6.88 ± 1.94, which was significantly higher than that in the other groups. Furthermore, the groups differed significantly in terms of TMD index. Interestingly, almost all TMD indexes, including neck PI, PI, and CMI, were significantly higher in the wTMD group than in the pTMD and iTMD groups (Table 1). In contrast, the mean DI value did not differ among the groups, nor did the mean values of CMO, MMO, or eccentric movement.

### Clinical Characteristics in wTMD, pTMD, and iTMD Patients

Table 2 shows the putative contributing factors and clinical symptoms of TMD in each group. These data suggest that whiplash and TMD are correlated, and that this correlation was significantly stronger in the wTMD group than in the pTMD and iTMD groups. The distributions of tinnitus, headache, and stress in the wTMD group were different from those in the other groups. Specifically, although stress was at its lowest level in the wTMD group (31.58%), wTMD patients were more likely to have tinnitus (32.89%), and headache (63.16%). Sleep problems were less common in the wTMD group, but not significantly (*p* = 0.059). Bruxism was significantly more prevalent in the iTMD group (17.07%) than in the other groups (*p* = 0.007); clenching was also more common (31.76%), but not significantly so (*p* = 0.326).

**Table 2:** Distribution of clinical findings and putative contributing factors for temporomandibular disorder (TMD).

Total (*n* = 219)	wTMD group *n* (%)	pTMD group *n* (%)	iTMD group *n* (%)	*p*-Value	*Post hoc* analysis
**TMJ noise**					
No	48 (63.16)	18 (31.03)	26 (30.59)	<0.0001	wTMD–pTMD
Yes	28 (36.84)	40 (68.97)	59 (69.41)		wTMD–iTMD
**TMJ pain**					
No	5 (6.58)	6 (10.34)	10 (11.76)	0.531	n.s.
Yes	71 (93.42)	52 (89.66)	75 (88.24)		
**Mouth opening limitation**					
No	59 (77.63)	40 (68.97)	63 (74.12)	0.526	n.s.
Yes	17 (22.37)	18 (31.03)	22 (25.88)		
**Bruxism**					
No	69 (90.79)	52 (89.66)	71 (83.53)	0.326	n.s.
Yes	7 (9.21)	6 (10.34)	14 (17.07)		
**Clenching**					
No	69 (90.79)	47 (81.03)	58 (68.24)	0.007	wTMD–iTMD
Yes	7 (9.21)	11 (18.97)	27 (31.76)		pTMD–iTMD
**Tinnitus**					
No	51 (67.11)	40 (68.97)	60 (70.59)	0.893	n.s.
Yes	25 (32.89)	18 (31.03)	25 (29.41)		
**Headache**					
No	28 (36.84)	32 (55.17)	42 (49.41)	0.087	n.s.
Yes	48 (63.16)	26 (44.83)	43 (50.59)		
**Stressful condition**					
No	52 (68.42)	26 (44.83)	42 (49.41)	<0.0001	wTMD–pTMD
Yes	24 (31.58)	32 (55.17)	43 (50.59)		wTMD–iTMD, pTMD–iTMD
**Sleep problem**					
No	75 (96.68)	52 (89.16)	81 (95.29)	0.059	n.s.
Yes	1 (1.32)	6 (10.34)	4 (4.71)		

*p-Value was obtained from χ^2^ test and the mean difference between groups was obtained by post hoc analysis with Boneferroni test. p-Value significance was set at <0.05*.

*wTMD–pTMD, when the proportion of the presence of variable was significantly different between wTMD and pTMD groups; wTMD–iTMD, when the proportion of the presence of variable was significantly different between wTMD and iTMD groups; pTMD–iTMD, when the proportion of the presence of variable was significantly different between pTMD and iTMD groups; n.s., when there was no significant difference between groups; TMJ, temporomandibular joint*.

### Distribution of MRI Findings

The distributions of a number of MRI variables were significantly different between the wTMD and iTMD groups (Table 3). Effusion was present in 5.3% of the wTMD patients, which was the lowest proportion among the three groups. The highest effusion proportion occurred in the pTMD group (18.97%), but it was not significantly different from the iTMD (17.65%) in this regard. In addition, the wTMD group had significantly more changes than the iTMD group in terms of disk deformity and VC of the LPM, as measured using MRI. Conversely, the presence of ADDWoR was significantly higher in the iTMD group than in the wTMD group.

**Table 3:** Comparison between magnetic resonance imaging variables in groups measured categorically.

		wTMD group		pTMD group		iTMD group			
		*n* = 76	Column (%)	*n* = 58	Column (%)	*n* = 85	Column (%)	*p*-Value	*Post hoc* analysis
Effusion	No	72	94.74	47	81.03	70	82.35	0.029	wTMD–pTMD
	Yes	4	5.26	11	18.97	15	17.65		wTMD–iTMD
ADDWR	No	45	59.21	38	65.52	56	65.88	0.634	n.s.
	Yes	31	40.79	20	34.48	29	34.12		
ADDWoR	No	48	63.16	36	62.07	37	43.53	0.021	wTMD–pTMD
	Yes	28	36.84	22	37.93	48	56.47		pTMD–iTMD
Disk deformity	No	39	51.32	36	62.07	57	67.06	0.119	wTMD–iTMD
	Yes	37	48.68	22	37.93	28	32.94		
Condylar degeneration	No	44	57.89	36	62.07	52	61.18	0.867	n.s.
	Yes	32	42.11	22	37.93	33	38.82		
VC of LPM	No	39	51.32	36	62.07	57	67.06	0.049	wTMD–iTMD
	Yes	37	48.68	22	37.93	28	32.94		
SC of LPM	No	45	59.21	31	53.45	53	62.35	0.777	n.s.
	Yes	31	40.79	27	46.55	32	37.65		

*p-Value was obtained from χ^2^ test and the mean difference between groups was obtained from post hoc analysis with Boneferroni test. p-Value significance was set at <0.05*.

*ADDWR, anterior disk displacement with reduction; ADDWoR, anterior disk displacement without reduction; VC, volume change; SC, signal change; LPM, lateral pterygoid muscle; wTMD–pTMD, when the proportion of the presence of variable was significantly different between wTMD and pTMD groups; wTMD–iTMD, when the proportion of the presence of variable was significantly different between wTMD and iTMD groups; pTMD–iTMD, when the proportion of the presence of variable was significantly different between pTMD and iTMD groups; n.s., when there was no significant difference between groups*.

### Correlation Coefficients of the VAS Score, Clinical Symptoms of TMD, and MRI Variables

Visual analog scale was strongly correlated with both headache and stress in wTMD patients. In addition, there were strong bidirectional correlations among some MRI variables; specifically, ADDWR, ADDWoR, disk deformity, and condylar degeneration were significantly correlated with each other in all groups. With regard to the LPM-related MRI findings, significant positive correlations were found between the SC and VC of the LPM, and both SC and VC were significantly correlated with ADDWoR, disk deformity, and condylar degeneration in the wTMD and iTMD groups; these correlations were not observed in the pTMD group (Table 4). However, the VAS score, which indicates the degree of subjective pain, was not significantly associated with any of these MRI variables in wTMD or iTMD patients. In pTMD patients, neck PI was correlated with ADDWR and PI was correlated with effusion. There was no correlation between effusion and other clinical findings in the wTMD group. However, there was a positive correlation between effusion and neck PI in the pTMD group, and between TMJ pain and the VAS score in the pTMD and iTMD groups (Table SA1 in Supplementary Material).

**Table 4:** Evaluation of correlation coefficient (*r*) among the variables obtained from magnetic resonance imaging.

	wTMD (*n* = 76)						pTMD (*n* = 58)						iTMD (*n* = 85)					
*r*	SC of LPM	Effusion	ADDWR	ADDWoR	Disk deformity	Condylar degeneration	SC of LPM	Effusion	ADDWR	ADDWoR	Disk deformity	Condylar degeneration	SC of LPM	Effusion	ADDWR	ADDWoR	Disk deformity	Condylar degeneration
VC of LPM	0.424**	0.124	0.049	0.511**	1.000**	0.609**	−0.088	−0.016	−0.044	0.268*	1.000**	0.268*	0.385**	0.004	−0.029	0.464**	1.000**	0.520**
SC of LPM		0.044	0.183	0.476**	0.424**	0.323**		0.254	0.050	0.268*	−0.088	0.054		−0.041	−0.047	0.290**	0.385**	0.328**
Effusion			−0.076	0.186	0.124	0.157			−0.073	0.256	−0.016	−0.106			−0.008	0.344**	0.004	0.201
ADDWR				0.088	0.049	−0.057				−0.044	−0.044	0.106				0.031	−0.029	−0.013
ADDWoR					0.511**	0.564**					0.268*	0.414**					0.464**	0.553**
Disk deformity						0.609**						0.268*						0.520**

p-Value was considered as significant when p < 0.05 (*p < 0.05, **p < 0.01). When p-value was in the significant level, we bolded the number to highlight the results.

*VC, volume change; SC, signal change; LPM, lateral pterygoid muscle; ADDWR, anterior disk displacement with reduction; ADDWoR, anterior disk displacement without reduction; r, correlation coefficient which was produced by the Spearman’s correlation test*.

### Changes in the Odds Ratio of Variables Over Time

Odds ratios were calculated for the association between each TMD clinical factor and MRI findings, with adjustment for age and sex. When we analyzed the data as a whole, the odds ratios of (1) SC and VC of the LPM, (2) the ADDWR, and (3) condylar degeneration increased over time. In wTMD patients, a time-dependent increase in ADDWR was predicted in the logistic regression model. However, the MRI variables showed no tendency to increase over time, as was observed previously in the pTMD and iTMD patients (Table SA2 in Supplementary Material).

## Discussion

A common but unique trauma, whiplash is caused by hyperextension and hyperflexion of the cervical spine; head and neck pain are the most prominent symptoms in the acute and chronic phases of whiplash (18). As hypothesized, the present study demonstrated that many of the clinical symptoms and MRI findings of whiplash-related TMD can be distinguished from those of TMD resulting from another macrotrauma or from unknown causes. This may imply that patients with whiplash have different physiologic responses and pain control mechanisms. Previous studies have revealed that patients with whiplash injury report higher pain scores, longer pain duration, and larger areas of local and referred pain than healthy controls (19). This was consistent with our main finding that wTMD patients had higher pain intensity across wider jaw and neck areas compared with pTMD and iTMD patients. Interestingly, in the wTMD group, LPM changes were significantly associated with both disk displacement and condylar degeneration; such an association was not observed in the pTMD group. Finally, the odds ratios in the time-dependent increase pattern analysis suggested that whiplash injury contributes to the long-term effects of TMD.

The increases in clinical pain observed in the wTMD group may have been due to reductions in diffuse noxious inhibitory controls (DNICs), which inhibit pain control in central nervous system pain (20). Moreover, previous studies have reported that sleep fragmentation is related to decreased DNICs in patients with TMD (21); others have confirmed that changes in DNICs parallel changes in clinical pain, and that DNICs prospectively predict long-term post-surgical pain (22). Alternatively, chronic whiplash is associated with reduced reactivity and enhanced negative feedback suppression in the hypothalamic–pituitary adrenal (HPA) axis. Furthermore, in clinical studies, HPA axis dysfunction has been associated with chronic widespread body pain (23). Collectively, changes to the process of central endogenous pain inhibition in patients with whiplash-related TMD may interfere with DNICs or the HPA axis; therefore, clinical pain is more likely to be amplified in such patients.

Decreased pain thresholds or sensory hypersensitivity have been demonstrated both locally, throughout the neck region, and at more distant or remote sites where there is no damage (24). Whiplash injury damages the deep tissues of the facet joint by compression and/or stretching; associated shear forces harm the disk, and can affect the TMJ. This suggests that pain hypersensitivity is not limited to the injured area, and that central sensitization of nociceptive pathways is the cause of pain hypersensitivity in TMD. In this regard, central hypersensitivity as a determinant of widespread pain and increased initial pain intensity is probably a dynamic condition that is influenced by the presence and activity of a nociceptive pathway (25). Wind-up and central sensitization, which rely on central pain mechanisms, occur after prolonged C-nociceptor input and depend on the activation of nociceptor-specific and wide dynamic-range neurons in the dorsal horn of the spinal cord (26). Thalamic activity also contributes significantly to the pain processing. Decreased thalamic activity can cause exaggerated pain following low-intensity nociceptive or innocuous peripheral stimulation (27). There is now overwhelming data demonstrating that patients with whiplash-related TMD have sensory disturbances, including decreased pain thresholds after various stimuli (19). Importantly, however, central hypersensitivity is not specific to whiplash—it has been observed in different chronic pain syndromes, including TMJ pain (28). This suggests that similar processes may underlie various chronic pain conditions, and that the differences between these conditions require further investigation.

Unlike microtrauma, most macrotrauma is unexpected and results from the sudden delivery of great force that can change structure. When this occurs, the teeth are separated, resulting in injury to the joint structures. Unexpected macrotrauma to the jaw can lead to disk displacement (3). The most common type of indirect macrotrauma is associated with whiplash injury (2, 29). Although the literature does report that whiplash injury is associated with TMD symptoms, data regarding the precise nature of this relationship are still lacking. One computer model suggested that certain motor vehicle injuries do not produce a TMJ flexion–extension event similar to that seen in the neck (30), and it is not certain whether such an event happens in whiplash, especially when the injury occurs at low velocity.

Alterations to the LPM and disk displacement due to whiplash injury can be a key risk factor for TMD. In the present study, we also found that disk displacement was significantly correlated with LPM changes, although this was true only in the wTMD and iTMD groups. The LPM contributes to jaw movement control through its attachment to the TMJ disk and condyle. In fact, the muscle appears to be involved in mandibular protrusion and lateral movement; in the latter, it is assisted by the masseter, medial pterygoid, and temporal muscles. The LPM is also important to the functioning of the TMJ disk (31). In particular, the superior head influences rotation and translation of the disk and condyle, and internal derangement is related to temporomandibular dysfunction (31). Taken together, we can clearly see why LPM, disk displacement, and condylar degeneration are strongly related to whiplash injury. Inflammation of the TMJ and retrodiscal tissue, effusion, and disk displacement can be explained in terms of changes to the LPM (29). However, D’Ippolito et al. reported that patients without TMD can also present with changes in LPM thickness, such as atrophy and contracture in the TMJ images (5). Contraction of the muscle related to spasm and/or edema likely increases the thickness of the muscle belly, whereas atrophy and fat infiltration may lead to a relative decrease in thickness, which would be measurable on MRI. Future studies should focus on the biochemical changes to the LPM after whiplash injury.

In addition to the mechanisms presented above, the TMJ may be indirectly influenced by whiplash injury. Neck injury is associated with disturbed control of mandibular and head–neck movements during jaw opening–closing tasks; therefore, such injuries can compromise natural jaw function (32). Furthermore, jaw and head–neck movements have common neural networks and commands that are pre-programmed reactions (29). Whiplash injury causes damage to the deep tissue of the facet joint by compression and/or stretching, as well as to the disk by shear forces. Furthermore, the cervical facet capsular ligaments can be injured in rear-end impacts by combined shear force, bending, and compression. In particular, the cervical facet capsular ligaments can be injured under loading conditions similar to those generated during whiplash (8 km/h rear-end collision) (33). Taken together, neck trauma is associated with disturbed jaw–neck function, and that jaw function in particular can be compromised in whiplash injury. Specifically, jaw functions such as gaping, biting, chewing, swallowing, yawning, and communication can be hampered after neck trauma (34). The neural networks controlling concomitant mandibular and head–neck movements during jaw function extend caudally in the brainstem and involve cervical spine segments. These networks jaw and neck muscle synergies to be created with central commands in common. Therefore, we propose that future research into the central mechanism behind jaw function should address head–neck motor control and involvement of the cervical spine.

Psychological factors, as well as physical factor, can play a role in the progress or recovery from whiplash injury (35). We observed that increased TMD pain intensity was associated with headache and psychological distress in the wTMD group, and the prevalence of headache was higher than in the other groups. Psychological trauma can differ from other types of injury. The interconnectivity between psychological and biological factors can involve the development, processing, consequences, and chronicity of headache (36). Brain regions associated with pain processing are also related with other psychological phenomenon, therefore, modulation of headache pain and changes of psychological factors may evoke through shared brain circuits, modifying the pain signal within the brain (37). Headache has been suggested as an aggravating and potential risk factor for TMD symptoms (9, 38). In addition, pain catastrophizing can be a potent predictor of pain intensity and psychological distress, and it is not dependent on the level of physical impairment (39). In addition, sleep problems also deleteriously affect central pain-modulatory systems (20). In the wTMD group of the present study, sleep problems were correlated with PI scores only. Therefore, we would suggest that patients with whiplash-related TMD have unstable coping strategies and that they catastrophize pain.

The mean time from symptom onset to diagnosis of wTMD patients was 190.09 ± 502.28 days, which was significantly shorter than that of pTMD (691.38 ± 1,573.04 days) and iTMD patients (669.82 ± 1,286.21 days). Perhaps this was because both insurance coverage and symptom severity are greater after car accidents than after other traumas or microtrauma. Nonetheless, the difference in the period between onset and diagnosis could be considered a noteworthy clinical feature of patients with whiplash-related TMD. The discrepancy may occur because patients with whiplash-related TMD have more severe symptoms as a result of the massive impact, and because car insurance generally covers medical expenses. As far we know, the present study was the first to compare the clinical and MRI features between patients with whiplash-related TMD and those with TMD caused by another trauma or no trauma. Because asymptomatic patients became symptomatic after whiplash injury, it is likely that TMJ disorders are related to whiplash.

### Conclusion

In the present study, the wTMD group had significantly higher initial pain levels than both the pTMD and iTMD groups. Moreover, LPM alterations were significantly related to disk displacement, effusion, and condylar degeneration in wTMD patients, but not in the pTMD group. The estimated odds ratio of ADDWR increased significantly over time in the wTMD group only. The distinctive features of our wTMD group may suggest that whiplash injury is associated with TMD, and they may indicate the possible mechanisms of this relationship. In addition, if clinicians can learn to recognize the LPM alterations that occurred in our wTMD group, they will better understand the clinical symptoms and pathophysiology of whiplash-related TMD. Researches must explore how altered neuromuscular, neuropsychological, and central pain control relates to TMD pain or symptoms. Importantly though, whiplash injury and TMD are not homogenous conditions. Therefore, in cases of whiplash injury, clinicians should always remember that the pathogenesis of traumatic TMD differs from that of non-traumatic TMD.

## Ethics Statement

Ethics approval and consent to participate—procedures on human subjects are done in accord with the ethical standards of the Committee on Human Experimentation of our institution in accord with the Helsinki Declaration of 1975. In addition, the study design in the manuscript was approved by the appropriate ethics review boards of Kyung Hee University Dental Hospital. Consent for publication—the authors had all responsibilities for the content and writing of the paper.

## Author Contributions

Y-HL and KL substantial contributions to conception and design, acquisition of data, or analysis and interpretation of data. Q-SA and J-PH contributions to acquisition of data, or analysis and interpretation of data. Y-HL and KL drafting or revising of the manuscript critically for important intellectual content. Final manuscript approval for submission and publication obtained from all authors.

## Conflict of Interest Statement

The authors declare that the research was conducted in the absence of any commercial or financial relationships that could be construed as a potential conflict of interest.
