# Supplementary material for: Magnetic Resonance Imaging-Based Prediction of the Relationship between Whiplash Injury and Temporomandibular Disorders
Source: Front Neurol. 2018 Jan 9;8:725. doi: 10.3389/fneur.2017.00725 (PMC5767220; doi:10.3389/fneur.2017.00725)
Supplement: Supplementary file 1 [file Data_Sheet_1.DOCX]

**Appendix table 1. Correlation coefficient (r) among the clinical variables and MRI findings**

| **r** | **Clinical findings** | | | | | | | | | |  | **MRI findings** | | | | | | |
| --- | --- | --- | --- | --- | --- | --- | --- | --- | --- | --- | --- | --- | --- | --- | --- | --- | --- | --- |
| **TMD index** | **VAS** | **TMJ noise** | **TMJ pain** | **Mouth opening limitation** | **Bruxism** | **Clenching** | **Tinnitus** | **Headache** | **Stressful condition** | **Sleep problem** |  | **Effusion** | **ADDWR** | **ADDWoR** | **Disc deformity** | **Condylar degeneration** | **VC of LPM** | **SC of LPM** |
| **wTMD group** | |  |  |  |  |  |  |  |  |  |  |  |  |  |  |  |  |  |
|  |  |  |  |  |  |  |  |  |  |  |  |  |  |  |  |  |  |  |
| **VAS** | 1 | .050 | .119 | **.280^*^** | -.022 | -.215 | .193 | **.299^**^** | **.434^**^** | .071 |  | .145 | -.215 | -.052 | -.030 | -.013 | -.030 | -.002 |
| **Neck PI** | .092 | .006 | .032 | .074 | .071 | -.020 | .084 | .158 | -.003 | .125 |  | -.218 | .200 | -.049 | -.002 | -.039 | -.002 | .039 |
| **PI** | .182 | -.036 | -.002 | .130 | -.158 | -.044 | .005 | .105 | .090 | .233^*^ |  | -.117 | -.075 | .032 | -.020 | -.088 | -.020 | .194 |
| **DI** | .206 | .029 | -.025 | .038 | -.079 | -.171 | .179 | .136 | .213 | -.128 |  | -.025 | .047 | .084 | -.024 | -.197 | -.024 | .154 |
| **CMI** | **.374^**^** | .068 | .066 | .111 | -.021 | -.118 | .127 | **.284^*^** | .208 | .170 |  | -.033 | -.103 | .068 | -.039 | -.063 | -.039 | .127 |
| **pTMD group** | |  |  |  |  |  |  |  |  |  |  |  |  |  |  |  |  |  |
|  |  |  |  |  |  |  |  |  |  |  |  |  |  |  |  |  |  |  |
| **VAS** | 1 | .046 | **.261^*^** | .172 | .121 | .103 | .256 | -.006 | .115 | .045 |  | .084 | -.198 | -.089 | -.041 | **-.329^*^** | -.041 | -.032 |
| **Neck PI** | .147 | -.139 | -.071 | .017 | -.115 | -.020 | -.105 | .149 | .079 | -.115 |  | **.269^*^** | .111 | -.149 | -.032 | .084 | -.032 | .023 |
| **PI** | -.018 | -.077 | .064 | .077 | -.064 | -.091 | .077 | .020 | .170 | -.064 |  | .150 | .260^*^ | .047 | .047 | .047 | .047 | .202 |
| **DI** | .205 | .185 | .055 | .117 | .174 | -.049 | **.269^*^** | .228 | .124 | -.055 |  | .040 | -.094 | **-.296^*^** | -.152 | -.080 | -.152 | -.223 |
| **CMI** | .179 | -.192 | .104 | **.325^*^** | -.104 | .165 | .060 | .217 | .030 | -.104 |  | .165 | .165 | -.114 | .013 | .013 | .013 | -.040 |
| **iTMD group** | |  |  |  |  |  |  |  |  |  |  |  |  |  |  |  |  |  |
|  |  |  |  |  |  |  |  |  |  |  |  |  |  |  |  |  |  |  |
| **VAS** | 1 | -.077 | **.239^*^** | .209 | .035 | -.030 | .142 | **.263^*^** | .052 | .004 |  | .011 | .104 | -.128 | -.195 | .037 | -.195 | -.213 |
| **Neck PI** | .162 | -.002 | -.048 | .197 | -.101 | -.009 | .156 | **.355^**^** | **.224^*^** | -.094 |  | .061 | -.030 | .175 | -.020 | .131 | -.020 | .075 |
| **PI** | .176 | -.094 | -.091 | .122 | -.099 | -.032 | .222^*^ | **.220^*^** | **.220^*^** | -.049 |  | -.103 | -.043 | .083 | -.038 | .165 | -.038 | .057 |
| **DI** | .205 | .036 | .075 | **.267^*^** | -.008 | .059 | .207 | .173 | -.188 | .100 |  | .108 | .026 | .150 | -.068 | .175 | -.068 | -.129 |
| **CMI** | .210 | .019 | -.035 | .100 | .059 | .174 | .201 | **.249^*^** | .058 | .051 |  | .044 | -.052 | .134 | -.109 | .102 | -.109 | -.083 |

VAS: Visual analogue scale, PI: palpation index, DI: dysfunction index, CMI: craniomandibular index, ADDWR: anterior disc displacement with reduction, ADDWoR: anterior disc displacement without reduction, LPM: lateral pterygoid muscle, VC: volume change, SC: signal change

r: correlation coefficient, which was produced by the Spearman's correlation test. *p*-value was considered as significant when p-value < 0.05. (*: P<0.05, **: P<0.01) When *p*-value was in the significant level, we bolded the number to highlight the results.

| **Appendix table 2. Variation in the odds ratio of MRI findings over time**   \|  \| **1 day (Ref*)** \| **1 month** \|  \| **1 year** \|  \| **5 years** \|  \| **10 years** \|  \|  \| \| --- \| --- \| --- \| --- \| --- \| --- \| --- \| --- \| --- \| --- \| --- \| \| MRI variables \| **OR** \| **OR** \| **95% CI** \| **OR** \| **95% CI** \| **OR** \| **95% CI** \| **OR** \| **95% CI** \| ***p*-value** \| \| **Total** \|  \|  \|  \|  \|  \|  \|  \|  \|  \|  \| \| Effusion \| 1.000 \| 0.999 \| 0.990-1.009 \| 0.993 \| 0.888-1.109 \| 0.964 \| 0.561-1.657 \| 0.930 \| 0.315-2.744 \| 0.895 \| \| ADDWR **^§^** \| 1.000 \| 1.007 \| 1.000-1.014 \| 1.091 \| 1.001-1.189 \| 1.528 \| 1.004-2.327 \| 2.336 \| 1.008-5.413 \| 0.043 \| \| ADDWoR **^¥^** \| 1.000 \| 1.003 \| 0.996-1.010 \| 1.035 \| 0.947-1.132 \| 1.185 \| 0.766-1.833 \| 1.404 \| 0.587-3.360 \| 0.071 \| \| Disc deformity **^¥^** \| 1.000 \| 0.998 \| 0.990-1.006 \| 0.978 \| 0.888-1.076 \| 0.897 \| 0.562-1.432 \| 0.804 \| 0.316-2.050 \| 0.054 \| \| Degeneration **^§^** \| 1.000 \| 1.004 \| 0.997-1.011 \| 1.048 \| 0.962-1.142 \| 1.256 \| 0.828-1.907 \| 1.579 \| 0.685-3.638 \| 0.047 \| \| SC of LPM **^¥^** \| 1.000 \| 1.003 \| 0.996-1.010 \| 1.037 \| 0.952-1.131 \| 1.195 \| 0.785-1.819 \| 1.429 \| 0.617-3.310 \| 0.051 \| \| VC of LPM **^¥^** \| 1.000 \| 1.002 \| 0.995-1.009 \| 1.025 \| 0.941-1.117 \| 1.130 \| 0.744-1.716 \| 1.277 \| 0.554-2.945 \| 0.051 \| \| **wTMD group (n=76)** \| \|  \|  \|  \|  \|  \|  \|  \|  \|  \| \| Effusion \| 1.000 \| 0.73 \| 0.29-1.81 \| 0.02 \| <0.001 >999.999 \| <0.001 \| <0.001  >999.999 \| <0.001 \| <0.001  >999.999 \| 0.497 \| \| ADDWR **^¥^** \| 1.000 \| 1.07 \| 1.00-1.15 \| 2.23 \| 0.96-5.02 \| 50.12 \| 0.81  >999.999 \| >999.999 \| 0.66  >999.999 \| 0.063 \| \| ADDWoR \| 1.000 \| 1.01 \| 0.99-1.04 \| 1.18 \| 0.83-1.67 \| 2.21 \| 0.40-12.10 \| 4.89 \| 0.16-146.40 \| 0.360 \| \| Disc deformity \| 1.000 \| 1.00 \| 0.97-1.03 \| 0.97 \| 0.68-1.38 \| 0.86 \| 0.16-4.80 \| 0.74 \| 0.02-23.00 \| 0.866 \| \| Degeneration \| 1.000 \| 1.03 \| 0.99-1.06 \| 1.38 \| 0.89-2.14 \| 4.74 \| 0.55-40.77 \| 22.48 \| 0.30  >999.999 \| 0.156 \| \| SC of LPM \| 1.000 \| 1.01 \| 0.98-1.04 \| 1.15 \| 0.81-1.64 \| 1.98 \| 0.35-11.15 \| 3.90 \| 0.12-124.31 \| 0.441 \| \| VC of LPM \| 1.000 \| 1.00 \| 0.98-1.03 \| 1.04 \| 0.74-1.47 \| 1.22 \| 0.23-6.51 \| 1.49 \| 0.05-42.34 \| 0.816 \| \| **pTMD group (n=58)** \| \|  \|  \|  \|  \|  \|  \|  \|  \|  \| \| Effusion \| 1.000 \| 1.00 \| 0.99-1.01 \| 1.03 \| 0.89-1.18 \| 1.13 \| 0.58-2.22 \| 1.28 \| 0.33-4.93 \| 0.716 \| \| ADDWR \| 1.000 \| 1.01 \| 1.00-1.02 \| 1.07 \| 0.94-1.22 \| 1.39 \| 0.74-2.61 \| 1.93 \| 0.55-6.80 \| 0.303 \| \| ADDWoR \| 1.000 \| 1.01 \| 1.00-1.02 \| 1.07 \| 0.94-1.22 \| 1.38 \| 0.73-2.61 \| 1.91 \| 0.54-6.79 \| 0.319 \| \| Disc deformity \| 1.000 \| 1.00 \| 0.99-1.01 \| 0.98 \| 0.85-1.12 \| 0.89 \| 0.45-1.75 \| 0.78 \| 0.20-3.05 \| 0.724 \| \| Degeneration \| 1.000 \| 1.00 \| 0.99-1.02 \| 1.04 \| 0.91-1.19 \| 1.22 \| 0.63-2.35 \| 1.48 \| 0.40-5.52 \| 0.558 \| \| SC of LPM \| 1.000 \| 1.01 \| 0.99-1.02 \| 1.06 \| 0.93-1.20 \| 1.31 \| 0.71-2.42 \| 1.71 \| 0.50-5.85 \| 0.394 \| \| VC of LPM \| 1.000 \| 1.00 \| 0.99-1.01 \| 1.04 \| 0.92-1.18 \| 1.21 \| 0.65-2.24 \| 1.46 \| 0.43-4.99 \| 0.550 \| \| **iTMD group (n=85)** \| \|  \|  \|  \|  \|  \|  \|  \|  \|  \| \| Effusion \| 1.000 \| 0.99 \| 0.98-1.01 \| 0.92 \| 0.74-1.15 \| 0.68 \| 0.23-2.00 \| 0.46 \| 0.05-3.99 \| 0.478 \| \| ADDWR \| 1.000 \| 1.01 \| 1.00-1.02 \| 1.08 \| 0.95-1.22 \| 1.42 \| 0.76-2.66 \| 2.02 \| 0.58-7.08 \| 0.270 \| \| ADDWoR \| 1.000 \| 1.00 \| 0.99-1.01 \| 0.99 \| 0.86-1.15 \| 0.97 \| 0.47-1.99 \| 0.94 \| 0.22-3.97 \| 0.936 \| \| Disc deformity \| 1.000 \| 1.00 \| 0.99-1.02 \| 1.03 \| 0.89-1.19 \| 1.16 \| 0.57-2.37 \| 1.34 \| 0.32-5.59 \| 0.685 \| \| Degeneration \| 1.000 \| 1.01 \| 1.00-1.02 \| 1.07 \| 0.94-1.23 \| 1.42 \| 0.74-2.72 \| 2.02 \| 0.55-7.40 \| 0.291 \| \| SC of LPM \| 1.000 \| 1.00 \| 0.99-1.01 \| 1.02 \| 0.90-1.17 \| 1.12 \| 0.59-2.14 \| 1.26 \| 0.35-4.57 \| 0.725 \| \| VC of LPM \| 1.000 \| 1.00 \| 0.99-1.01 \| 1.03 \| 0.90-1.18 \| 1.16 \| 0.61-2.19 \| 1.34 \| 0.38-4.81 \| 0.651 \| \| §: when the mean value of variable is significantly different between wTMD and pTMD \| \| \| \| \| \| \| \| \| \| \| \| ¥: when the difference in the mean value of variable is not statistically insignificant, but close to being significant \| \| \| \| \| \| \| \| \| \| \| \| * Reference group used to calculate odds ratios \| \| \| \| \| \| \| \| \| \| \| \| ADDWR: anterior disc displacement with reduction, ADDWoR: anterior disc displacement without reduction, LPM: lateral pterygoid muscle, VC: volume change, SC: signal change, OR; odds ratio  Results were obtained via logistic regression analysis. \| \| \| \| \| \| \| \| \| \| \| \| *p*-value was considered as significant when *p*-value < 0.05. \| \| \| \| \| \| \| \| \| \| \| |  |  |  |
| --- | --- | --- | --- | --- | --- | --- | --- | --- | --- | --- | --- | --- | --- | --- | --- | --- | --- | --- | --- | --- | --- | --- | --- | --- | --- | --- | --- | --- | --- | --- | --- | --- | --- | --- | --- | --- | --- | --- | --- | --- | --- | --- | --- | --- | --- | --- | --- | --- | --- | --- | --- | --- | --- | --- | --- | --- | --- | --- | --- | --- | --- | --- | --- | --- | --- | --- | --- | --- | --- | --- | --- | --- | --- | --- | --- | --- | --- | --- | --- | --- | --- | --- | --- | --- | --- | --- | --- | --- | --- | --- | --- | --- | --- | --- | --- | --- | --- | --- | --- | --- | --- | --- | --- | --- | --- | --- | --- | --- | --- | --- | --- | --- | --- | --- | --- | --- | --- | --- | --- | --- | --- | --- | --- | --- | --- | --- | --- | --- | --- | --- | --- | --- | --- | --- | --- | --- | --- | --- | --- | --- | --- | --- | --- | --- | --- | --- | --- | --- | --- | --- | --- | --- | --- | --- | --- | --- | --- | --- | --- | --- | --- | --- | --- | --- | --- | --- | --- | --- | --- | --- | --- | --- | --- | --- | --- | --- | --- | --- | --- | --- | --- | --- | --- | --- | --- | --- | --- | --- | --- | --- | --- | --- | --- | --- | --- | --- | --- | --- | --- | --- | --- | --- | --- | --- | --- | --- | --- | --- | --- | --- | --- | --- | --- | --- | --- | --- | --- | --- | --- | --- | --- | --- | --- | --- | --- | --- | --- | --- | --- | --- | --- | --- | --- | --- | --- | --- | --- | --- | --- | --- | --- | --- | --- | --- | --- | --- | --- | --- | --- | --- | --- | --- | --- | --- | --- | --- | --- | --- | --- | --- | --- | --- | --- | --- | --- | --- | --- | --- | --- | --- | --- | --- | --- | --- | --- | --- | --- | --- | --- | --- | --- | --- | --- | --- | --- | --- | --- | --- | --- | --- | --- | --- | --- | --- | --- | --- | --- | --- | --- | --- | --- | --- | --- | --- | --- | --- | --- | --- | --- | --- | --- | --- | --- | --- | --- | --- | --- | --- | --- | --- | --- | --- | --- | --- | --- | --- | --- | --- | --- | --- | --- | --- | --- | --- | --- | --- | --- | --- | --- | --- | --- | --- | --- | --- | --- | --- | --- | --- | --- | --- | --- | --- | --- | --- | --- | --- | --- | --- | --- | --- | --- | --- | --- | --- | --- | --- | --- | --- | --- | --- | --- | --- | --- | --- | --- | --- | --- | --- | --- | --- | --- | --- | --- | --- | --- | --- | --- | --- | --- | --- | --- | --- | --- | --- | --- | --- | --- | --- | --- | --- | --- | --- | --- | --- | --- | --- | --- | --- | --- | --- | --- | --- | --- | --- | --- | --- | --- | --- | --- | --- | --- | --- | --- | --- | --- | --- | --- | --- | --- | --- | --- | --- |
